# Auxin signaling in the cambium promotes tissue adhesion and vascular formation during Arabidopsis graft healing

**DOI:** 10.1093/plphys/kiae257

**Published:** 2024-05-03

**Authors:** Phanu T Serivichyaswat, Abdul Kareem, Ming Feng, Charles W Melnyk

**Affiliations:** Department of Plant Biology, Swedish University of Agricultural Sciences, Ulls gränd 1, 765 51 Uppsala, Sweden; Department of Plant Biology, Swedish University of Agricultural Sciences, Ulls gränd 1, 765 51 Uppsala, Sweden; Department of Plant Biology, Swedish University of Agricultural Sciences, Ulls gränd 1, 765 51 Uppsala, Sweden; Department of Plant Biology, Swedish University of Agricultural Sciences, Ulls gränd 1, 765 51 Uppsala, Sweden

## Abstract

The strong ability of plants to regenerate wounds is exemplified by grafting when two plants are cut and joined together to grow as one. During graft healing, tissues attach, cells proliferate, and the vasculatures connect to form a graft union. The plant hormone auxin plays a central role, and auxin-related mutants perturb grafting success. Here, we investigated the role of individual cell types and their response to auxin during Arabidopsis (*Arabidopsis thaliana*) graft formation. By employing a cell-specific inducible misexpression system, we blocked auxin response in individual cell types using the *bodenlos* mutation. We found that auxin signaling in procambial tissues was critical for successful tissue attachment and vascular differentiation. In addition, we found that auxin signaling was required for cell divisions of the procambial cells during graft formation. Loss of function mutants in cambial pathways also perturbed attachment and phloem reconnection. We propose that cambial and procambial tissues drive tissue attachment and vascular differentiation during successful grafting. Our study thus refines our knowledge of graft development and furthers our understanding of the regenerative role of the cambium.

## Teaser

Auxin signaling in the cambial tissues promotes cell divisions and drives both tissue attachment and vascular differentiation during successful Arabidopsis grafting.

## Introduction

Regeneration in response to injuries is vital for the survival of organisms. Plants have a robust regenerative ability that rapidly and efficiently heals wounds and reconnects tissues following damage (Ikeuchi et al. 2019). Since ancient times, people have taken advantage of such regenerative abilities to join the shoot (scion) of one plant to the root (rootstock) of another plant through a technique known as grafting (Garner and Bradley 2013). Grafting has improved yields by combining superior properties such as plant size and stress tolerance into one plant (Garner and Bradley 2013). After the cutting and joining of tissues, a series of regenerative events occur including tissue adhesion, the formation of an undifferentiated cell mass called callus, and the differentiation of vascular tissues between the scion and rootstock (Jeffree and Yeoman 1983; Melnyk et al. 2015). Tissue adhesion is facilitated by cell-wall modifying enzymes (Notaguchi et al. 2020) and by the expansion of epidermal and cortical cells to fill gaps at the wound site (Melnyk et al. 2015; Matsuoka et al. 2016). Cell divisions occur and wound-induced callus forms that are driven by *WOUND INDUCED DEDIFFERENTIATION1* (*WIND1*) and are thought to arise from vasculature and pericycle cells (Iwase et al. 2011; Matsuoka et al. 2016; Ikeuchi et al. 2017). Cambial cells likely also play an important role during grafting: wounding induces divisions in the cambium and mutations in several genes related to cambium, including DNA BINDING WITH ONE FINGER (DOF) and ARABIDOPSIS NAC DOMAIN CONTAINING PROTEIN (ANAC) transcription factors, reduce wound healing efficiency (Melnyk et al. 2015; Matsuoka et al. 2021; Zhang et al. 2022). In addition, horticultural texts highlight the importance of correct cambial alignment for grafting success (Garner and Bradley 2013). Thus, multiple cell types likely play pivotal roles during grafting but their functions are neither well described nor well defined.

Procambium plays an important role in vascular development and during primary growth undergoes periclinal divisions to form cambium. Several genes and pathways contribute to procambium and cambium formation (Shi et al. 2019). *PHLOEM INTERCALATED WITH XYLEM* (*PXY*) and *WUSCHEL RELATED HOMEOBOX4* (*WOX4*) are important for differentiating xylem cells and cambium cells close to the xylem (Shi et al. 2019), whereas *SMAX1-LIKE5* (*SMXL5*) is important for differentiating phloem cells and cambium cells close to the phloem (Shi et al. 2019). Such vascular differentiation of the cambium is a hallmark of secondary growth and requires auxin signaling (Smetana et al. 2019; Mäkilä et al. 2023). Auxin is well known for its role in promoting vascular development and patterning (Wetmore and Rier 1963; Sachs 1969). Auxin relies on perception by the TRANSPORT INHIBITOR RESPONSE1/AUXIN SIGNALING F-BOX (TIR1/AFB) receptors which degrade the Auxin/INDOLE-3-ACETIC ACID INDUCIBLE (Aux/IAA) proteins to allow AUXIN RESPONSE FACTORs (ARFs) activation (Leyser 2017). One well known Aux/IAA protein, BODENLOS/IAA12 (BDL), blocks auxin signaling with a point mutation in its degradation domain to prevent bdl degradation and allow ARFs to remain sequestered (Hamann et al. 2002; Leyser 2017). Auxin plays an important role during graft formation and perturbing auxin response with *bdl* mutants or chemically inhibiting auxin transport blocks grafting (Melnyk et al. 2015; Matsuoka et al. 2016). Investigating how auxin and *bdl* affect grafting is difficult because *bdl* is constitutively expressed in many cell types and the mutation has pleiotropic effects, particularly on embryonic root formation (Hamann et al. 2002). Here, to overcome this challenge and to investigate the role of individual cell types during graft formation and their response to auxin, we employed a chemically inducible gene expression system to block auxin signaling in different Arabidopsis (*Arabidopsis thaliana*) cell types by misexpressing *bdl*. We found that auxin signaling in the cambial tissues is required for both tissue attachment, vascular formation, and promoting cell divisions. Loss of function mutations in cambium-related genes similarly perturbed tissue attachment and vascular reconnection. Together, our findings highlight the importance of cambium as a driver of vascular differentiation and tissue fusion during graft healing.

## Results

### Establishing a tissue-specific inducible *bdl* system

**Figure 1.**
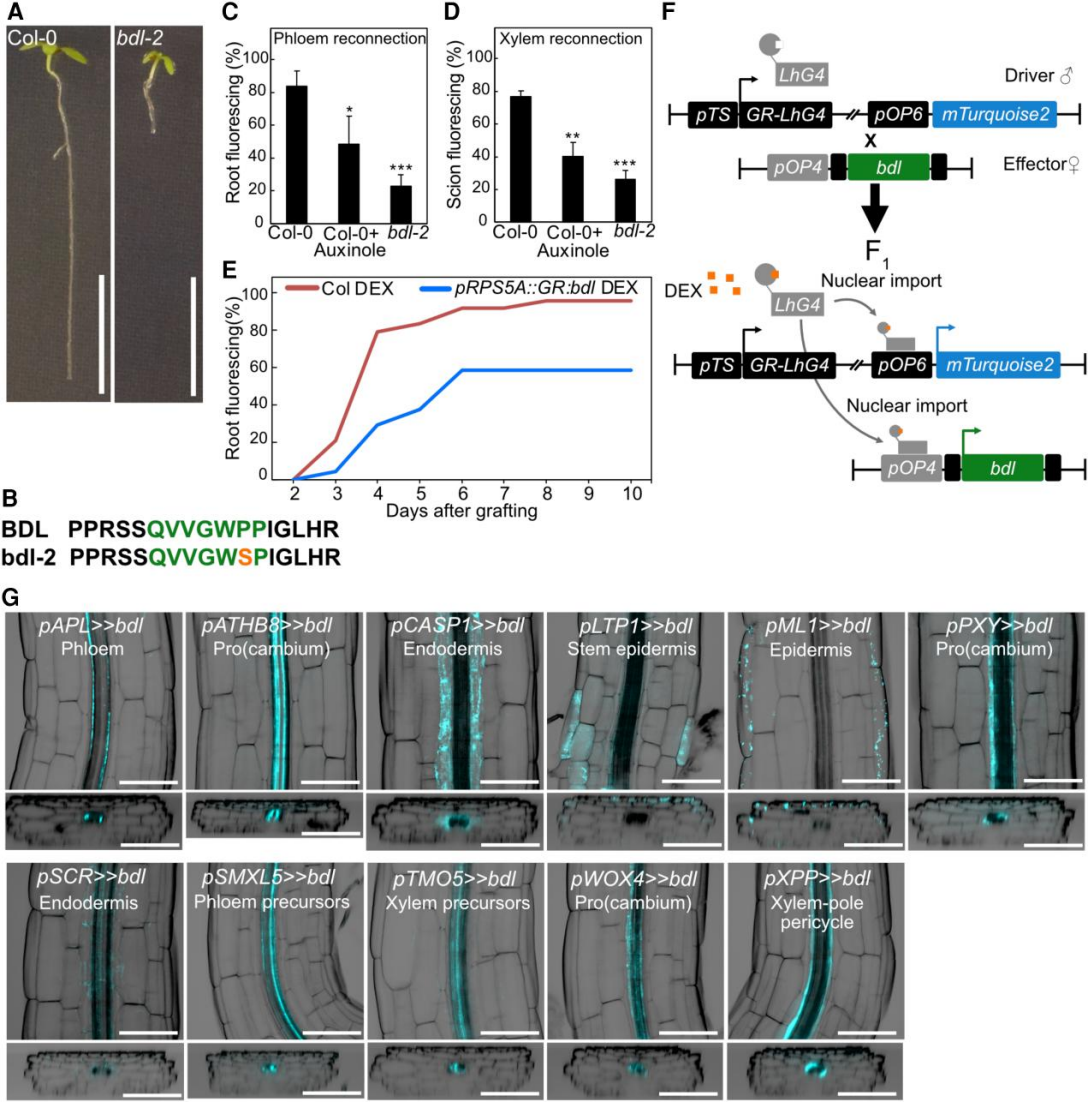
*bdl*-2 blocks graft formation and is useful in a cell-specific misexpression system. **A and B)***bdl* auxin resistance phenotype is caused by a point mutation resulting in a proline 74 to serine amino acid change in the conserved degradation domain. Scale bar is 1 cm. **C and D)** Proportion of grafted Arabidopsis that transported carboxyfluorescein diacetate (CFDA) to the rootstock at 4 d after grafting (DAG) **(C)**, or to the scion at 6 DAG **(D)**, 3 to 4 replicates, each with 8 to 16 plants for each grafted genotype or treatment. 30 μM auxinole was used. Values represent mean ± SD. *P < 0.05; **P < 0.01; ***P < 0.001; Student's *t*-test compared to mock controls. **E)***pSUC2::GFP* scion grafted to Col-0 or *GR:bdl* upon dexamethasone (DEX) treatment. Two replicates with 12 plants per time point per treatment. **F)** The design of inducible cell-type specific expression system. The *bdl* effector line was crossed with driver lines expressing a synthetic transcription factor GR-LhG under various tissue-specific promoters (*pTS*). In the resulting F_1_ seedlings, GR-LhG drives the expression of *bdl* as well as the *mTurquoise2* reporter when induced by DEX. **G)** Validation of tissue-specific expression of bdl in the targeted tissues by confocal imaging and detection of mTurquoise2 signals in the hypocotyl of 7-d-old seedlings. The images presented as longitudinal (top panels) and transverse (bottom panels) optical sections spanning through outer to inner tissue layers. The LUT for the calcofluor white signal, which marked the cell boundary, was modified to an inverted LUT in the Fiji software for better contrast. Scale bar is 100 *μ*m.

Auxin response is important for successful graft formation (Melnyk et al. 2015) and we tested this by treatment with the auxin receptor inhibitor auxinole and by grafting with the *bdl-2* mutant. Both reduced phloem and xylem reconnection at the graft junction (Fig. 1, A to D). However, the native *BDL* promoter drives *bdl* expression broadly in the stele (Hamann et al. 2002) suggesting that any one of these cell types might be relevant for graft formation. Previous studies have used an inducible *pRPS5A::GR:bdl* line (Weijers et al. 2006) and we found that this line reduced grafting efficiency (Fig. 1E) yet the *RPS5A* promoter expresses in many meristematic cell types and is not cell-type specific. To better understand where auxin signaling is required, we employed an inducible cell-specific expression system (Schürholz et al. 2018). We cloned the *bdl-2* mutant allele and drove it under a synthetic promoter (“effector line”) that activates only in the presence of a cognate transcription factor and crossed the resulting transgenic plant into 11 previously published lines (Schürholz et al. 2018) that express the transcription factor in an inducible and cell-specific manner (“driver line”) (Fig. 1F, Supplementary Fig. S1, A to C). Upon dexamethasone (DEX) induction of the resulting F1 plants, we observed tissue-specific mTurquoise2 fluorescence (Fig. 1G) consistent with cell-specificity of the transgene. DEX treatment increased hypocotyl lengths in plants expressing *bdl* in the procambium (*ARABIDOPSIS THALIANA HOMEOBOX FACTOR 8* (*pATHB8*), *pPXY*, *pWOX4*), xylem pole pericycle (*pXPP*), and developing phloem including procambium (*pSMXL5*) (Supplementary Fig. S2, A and B). Primary root lengths were not substantially changed but lateral root numbers decreased when *bdl* was misexpressed in the endodermis (*SCARECROW*, *pSCR*), phloem precursors (*pSMXL5*), procambium (*pATHB8*, *pPXY*, *pWOX4*), xylem pole pericycle (*pXPP*), and xylem precursors (*TARGET OF MONOPTEROS 5*, *pTMO5*) (Supplementary Fig. S2, C and D). Such effects were consistent with perturbed auxin signaling similar to what has been observed in other auxin signaling mutants (Leyser 2017; Reed et al. 2018).

### Auxin response in the procambium is required for successful graft formation

**Figure 2.**
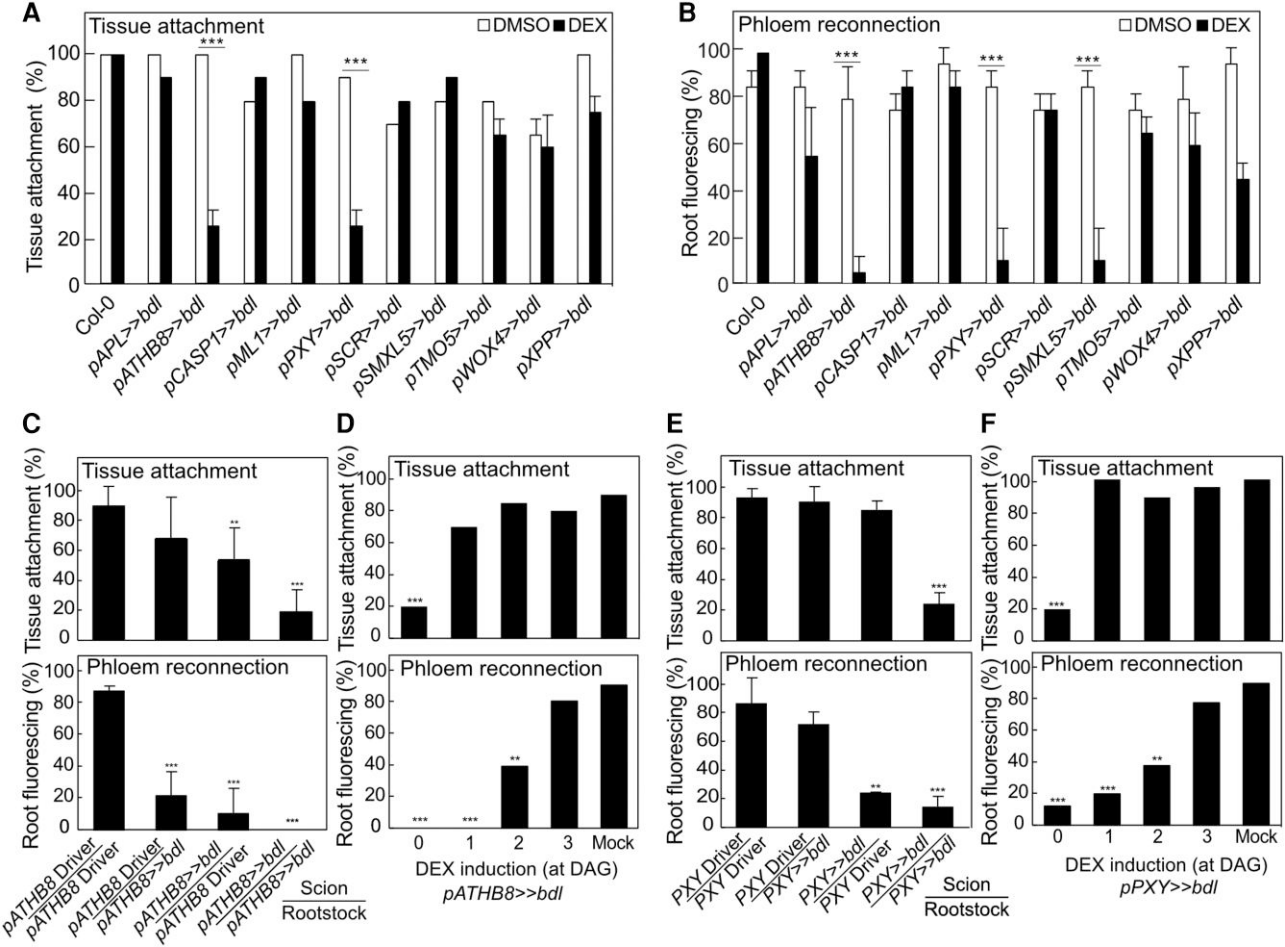
Perturbing auxin responses in the procambium inhibits successful graft formation. **A)** Proportion of grafts attached in transgenic Arabidopsis misexpressing *bdl* at 5 d after grafting (DAG). **B)** Proportion of grafted transgenic Arabidopsis that transported carboxyfluorescein diacetate (CFDA) to rootstocks at 5 DAG. **C and E)** Proportion of dexamethasone (DEX)-treated and grafted *pATHB8>>bdl***(C)** or *pPXY>>bdl***(E)** to the respective driver lines that attached or transported CFDA. The homo- and hetero-graft combinations are indicated. All plants were sampled at 5 DAG. **D and F)** Proportion of grafts attached or that transported CFDA to the rootstock of grafted *pATHB8>>bdl***(D)** or *pPXY>>bdl***(F)**. DEX was applied at indicated time points. Plants were sampled at 5 DAG. **A to F)***n* = 20 to 30 per treatment. Values represent mean ± SD. *P < 0.05; **P < 0.01; ***P < 0.001; Fisher's exact test compared to mock or driver controls.

Successful grafting involves tissue attachment followed by callus formation, phloem reconnection, and xylem reconnection (Melnyk et al. 2015). To understand the role of individual cell types in these processes, we performed tissue attachment and phloem reconnection assays on the misexpression lines. Plants expressing *bdl* in the procambium (*pATHB8* and *pPXY*) did not attach, form wound-induced callus, reconnect phloem, nor reconnect xylem (Fig. 2, A and B, Supplementary Fig. S3, A and B). However, *bdl* expression in the xylem pole pericycle cells (*pXPP*) and phloem precursors (*pSMXL5*) only inhibited phloem reconnection (Fig. 2, A and B, Supplementary Fig. S3, A and B). To exclude the possibility that this effect was specific to *bdl*, we also misexpressed a dominant mutant of the AUX/IAA gene, *iaa18*, and found similar effects on plant growth and a reduction in phloem reconnection by procambium and phloem precursor misexpression (Supplementary Figs. S3C and S4, A to D). Given the strong effects of *bdl* upon grafting, we tested the importance of *bdl* misexpression in the scion compared to the rootstock. While *pATHB8* driven *bdl* expression in the rootstock and scion reduced phloem reconnection, *pPXY* driven *bdl* expression only affected phloem reconnection when in the scion (Fig. 2, C and E). *pATHB8* driven *bdl* expression in the scion inhibited tissue attachment, while *pATHB8* and *pPXY* driven *bdl* expression in both rootstock and scion resulted in the strongest block in tissue attachment (Fig. 2, C and E). *pSMXL5* driven *bdl* expression had no effect on tissue attachment but blocked phloem reconnection when grafted as either the rootstock or the scion (Supplementary Fig. S5A). Given that *pPXY* driven *bdl* heterografts perturbed phloem reconnection despite normal attachment, this suggested that cambium had different roles during attachment and vascular formation. To further test the requirements of cambium, we grafted plants and then induced *bdl* for various days after grafting. Blocking auxin responses in procambium from 0 to 1 d after grafting (DAG) blocked attachment. Blocking auxin response 1 to 2 DAG allowed tissue attachment but inhibited phloem reconnection (Fig. 2, D and F). However, induction at 3 DAG in the cambium had no effect on tissue attachment or phloem reconnection, likely because phloem reconnections were forming by then (Fig. 2D and Supplementary Fig. S5B). Together, our data revealed a role for auxin signaling in the procambium driving tissue attachment and phloem reconnection through distinct mechanisms.

### Auxin responses are required for cell expansion and cell division during grafting

**Figure 3.**
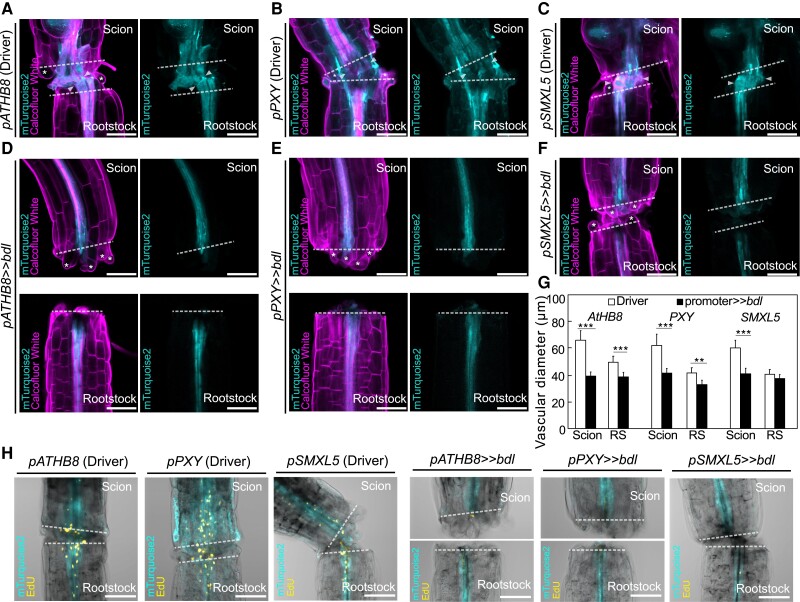
Auxin response is important for cambial expansion and cell divisions. Longitudinal optical sections of dexamethasone (DEX)-treated grafted hypocotyl driver lines **(A to C)** and *bdl* misexpression **(D to F)** at 5 d after grafting (DAG). **A to F)** Genotypes are indicated and white asterisks indicate expanding cortex and epidermal cells. White arrow heads indicate vascular connection in the graft junction. Dashed lines indicate the cut site; scale bars = 100 *µ*m. **G)** Vascular diameter including pericycle, cambium, xylem, and phloem of grafted scion or rootstock (RS) of indicated genotype at 5 DAG, 100 *µ*m from the cut surface (mean ± SD; *n* = 11 to 16 plants per genotype. *P < 0.05; **P < 0.01; ***P < 0.001; Student's *t*-test compared to driver lines). **H)** 5-Ethynyl-2′-deoxyuridine (EdU) staining detecting DNA replication at graft junctions of plants expressing *bdl* in various cell types at 3 DAG. The genotypes are indicated. Dashed lines indicate the cut site; scale bars = 100 *µ*m.

**Figure 4.**
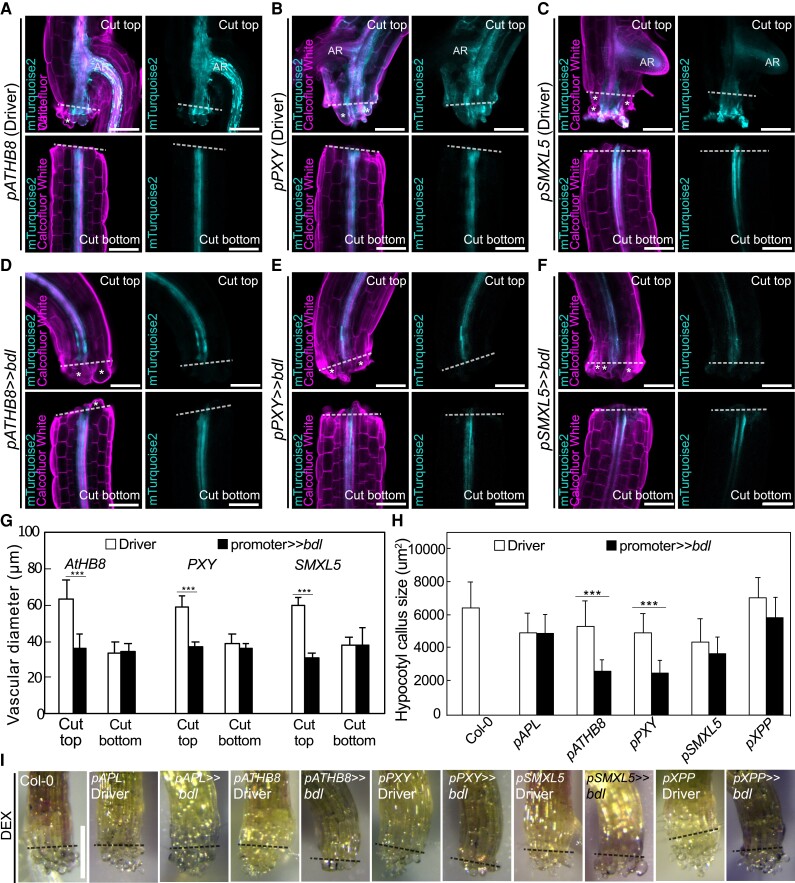
Auxin responses in cambium are required for tissue expansion and callus formation. Longitudinal optical sections of dexamethasone (DEX)-treated and cut but ungrafted hypocotyl driver lines **(A to C)** and *bdl* misexpression lines 5 d after cutting **(D to F)**. The genotypes are indicated. White asterisks indicate expanding cortex and epidermal cells. AR, adventitious root. Dashed lines indicate the cut site; bars = 100 *µ*m. **G)** Vascular diameter including pericycle, cambium, xylem, and phloem of cut but ungrafted plants of indicated genotype, 100 *µ*m from the cut surface (mean ± SD; *n* = 15 to 18 plants per genotype. *P < 0.05; **P < 0.01; ***P < 0.001; Student's *t*-test compared to driver lines). All plants were sampled at 5 d after cutting. **H)** Hypocotyl callus size from selected genotypes 5 d after cutting. Mean ± SD, *n* = 20 to 22 hypocotyl per genotype and treatment. *P < 0.05; **P < 0.01; ***P < 0.001; Student's *t*-test compared to mock controls. **I)** Callus formation from hypocotyls of cut but ungrafted driver lines or *bdl* lines treated with DEX. Scale bars = 500 *µ*m. Dashed lines indicate the cut site.

To understand how *bdl* blocked graft formation, we examined graft junctions misexpressing *mTurquoise2* with *pATHB8*, *pSMXL5*, and *pPXY* in the presence or absence of *bdl*. In driver lines alone, we observed *pATHB8* and *pSMXL5* drove *mTurquoise2* expression in the vasculature and the callus tissues connecting the scion and rootstock (Fig. 3, A and C). *pPXY* driven expression was more diffuse and appeared in the vasculature but also outer cell layers around the graft junction including the endodermis and cortex layers (Fig. 3B). In plants expressing both driver and *bdl* effector, *mTurquoise2* signal was noticeably reduced with little signal present at the cut site and the vascular diameters reduced (Fig. 3, D to G). *pSMXL5* driven *bdl* plants remained attached but this occurred through non-*SMXL5* expressing cells, likely instead cortex or epidermal tissues (Fig. 3F). To analyze the role of cell division, we stained with the fluorescent labeled thymidine-analogue EdU that incorporates into newly synthesized DNA of dividing cells (S phase) (Salic and Mitchison 2008). We detected high EdU staining in grafted driver tissues, particularly in the vasculature, consistent with high cell division in these tissues (Fig. 3H). Conversely, EdU signals were undetectable in the graft junction of plants expressing *bdl* in procambium and phloem precursors (Fig. 3H), implying that *bdl* suppressed cell division. Moreover, grafted plants expressing *bdl* in *pSMXL5* phloem precursors attached their tissues but with little cell division and no vascular connection (Fig. 3H). Our data suggested that graft failures induced by *bdl* expression were due in part to the inhibition of procambial cell division that prevented callus and phloem formation. To better observe the effects of *bdl* misexpression at the cut site, we imaged cut but non-grafted hypocotyls. Expression patterns for *pATHB8*, *pSMXL5*, and *pPXY* driver lines in cut tops were similar as those in the scion, but expression patterns in cut roots showed little cell division and little cell expansion compared to the grafted rootstock (Fig. 4, A to C and G). Thus, it appeared that contact between rootstock and scion was necessary for vascular proliferation in the rootstock. In cut but ungrafted plants expressing both driver and *bdl* effector, the *mTurquoise2* expression domain was greatly reduced and limited to the vascular tissues which showed little proliferation and expansion compared to the driver lines (Fig. 4, D to G). Hypocotyl callus formation from cut shoots was also reduced by *pATHB8* and *pPXY* driven *bdl* (Fig. 4, H and I), consistent with *bdl* misexpression in the cambium blocking vascular proliferation, cell expansion and callus formation.

**Figure 5.**
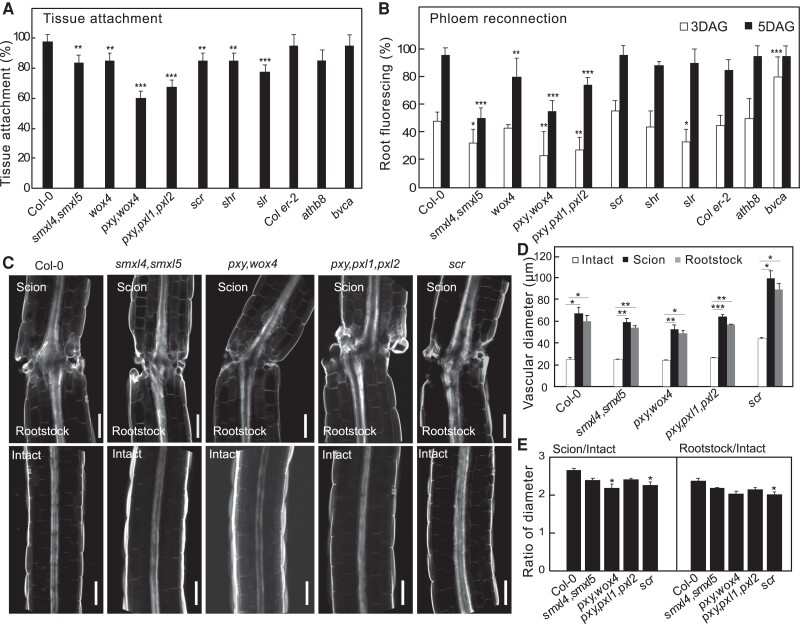
Cambial mutants are impaired in tissue attachment and phloem reconnection. **A)** Tissue attachment in wildtype and mutant Arabidopsis 2 d after grafting (DAG) (mean ± SD; *n* = 8 to 20 plants per replicate per genotype; *P< 0.05, **P < 0.01, ***P < 0.001, Fisher's exact test compared to wildtype). **B)** Phloem reconnection in wildtype and mutant Arabidopsis 3 and 5 DAG (mean ± SD; *n* = 8 to 20 plants per replicate per genotype; *P < 0.05, **P < 0.01, ***P < 0.001, Fisher's exact test compared to wildtype). **C)** Longitudinal optical sections of grafted or intact Arabidopsis 7 DAG. Scale bar is 100 *µ*m. **D and E)** Vascular diameters in wildtype and mutant Arabidopsis 7 DAG measured 50 *µ*m from the cut site. Measurements include pericycle, cambium, xylem, and phloem. Diameter ratios were calculated by dividing scion or rootstock diameter by the intact diameter of the same genotype (mean ± SD; *n* = 9 to 13 plants per replicate per genotype; *P < 0.05, **P < 0.01, ***P < 0.001, one-way ANOVA followed Tukey HSD test compared to intact **(D)** or compared to Col-0 **(E)**).

Our *bdl* misexpression analysis suggested an important role for the procambium and phloem precursors in graft attachment and phloem reconnection. To further investigate these roles, we grafted mutants associated with these cell types including cambium (*wox4*; *pxy,wox4*; *pxy,pxl1,pxl2*; *athb8*; *athb8,can,phb,phv (bvca)*), phloem precursors (*smxl4,smxl5*), xylem pole pericycle (*slr1*), or endodermis (*scr*; *shr*). Most higher order cambium-related mutants that impair cambium function also strongly reduced tissue attachment and phloem reconnection, though the *bvca* mutant that increases vascular cell numbers (Carlsbecker et al. 2010) increased reconnection (Fig. 5, A to C). *smxl4,smxl5* strongly reduced phloem reconnection whereas *scr*, *shr*, and *slr1* slightly reduced attachment rates and had minor effects upon phloem reconnection (Fig. 5, A to C). Grafting induced an increase in vascular diameters but only *pxy,wox4* and *scr* reduced vascular diameter ratios compared to wild type (Fig. 5, D and E). Together, these losses of function mutant data were consistent with our *bdl* misexpression analysis revealing a critical role for the cambium.

## Discussion

Successful graft formation employs multiple cellular regeneration processes including tissue adhesion, callus formation, and vascular reconnection. Here, we used an inducible cell-specific expression system to investigate the role of individual cell types during graft formation in a spatio-temporal and nondestructive manner. We observed that perturbing auxin signaling in procambium during graft formation inhibited cell division, graft attachment, callus formation, phloem differentiation, and xylem differentiation. Procambium appeared to have separate roles for attachment and vascular differentiation since we could allow attachment to occur yet block phloem and xylem reconnection through inducible *bdl* activation in the procambium. Thus, our data suggest that auxin signaling in the procambium was critical for attachment and phloem reconnection, and we propose that a subset of cambial cells gives rise to one process or the other. One set of *PXY* and *ATHB8* expressing cells may contribute to attachment and wound-induced callus, while a different set of *SMXL5*, *ATHB8*, and *PXY* expressing cells may contribute to vascular formation. Notably, *pSMXL5* driven *bdl* plants could attach and reconnect xylem (Fig. 2A and Supplementary Fig. S3) despite their inability to reconnect phloem (Fig. 2B), suggesting that phloem reconnection is not required for xylem reconnection, and auxin signaling in the *SMXL5* domain was not necessary for tissue attachment. We observed some spreading of these domains outside of the cambial area, particularly for *pPXY*, and our analysis is also limited by where the promoter is active and the strength of the promoter. However, the loss of function cambium mutants showed a reduction in attachment and phloem transport rates, consistent with the *bdl* analysis and consistent with a previous study reporting the requirement of auxin signaling for procambium differentiating into phloem precursors during plant radial growth (Smetana et al. 2019). *PXY* gene expression also maintains the polarity of cambial cell divisions by programing the cells into the phloem lineage during vascular development (Fisher and Turner 2007; Smit et al. 2019). Our analysis revealed that procambium (*pATHB8*), developing phloem including procambium on the phloem side (*pSMXL5*), and procambium on the xylem side (*pPXY*) impaired grafting when auxin signaling was blocked, yet notably, other procambium markers (*pWOX4*) or developing xylem (*pTMO5*) misexpressing *bdl* showed no effect. It's possible that these domains are not affected by a block in auxin signaling or show weaker effects due to redundancy with other domains or a weaker promoter. The *pWOX4* driven *bdl* and *iaa18* lines had less change in root and hypocotyl morphology compared to other cambial markers (Supplementary Figs. S2 and S4) consistent with a weaker overall phenotype in this line.

Our analyses not only revealed auxin responsive cell types but also shed light on the importance of rootstock or scion specific response. While *pATHB8* and *pSMXL5* driven *bdl* expression affected both rootstocks and scions, *pPXY* driven *bdl* expression had its strongest effects in the scion. Previous studies have shown that mutating auxin-related genes such as *ALF4*, *AXR1*, and *BDL* all have their strongest effects in the rootstock, while auxin biosynthesis genes such as YUCCAs are required in the scion (Melnyk et al. 2015; Serivichyaswat et al. 2022). These findings suggest that where auxin is perceived in the scion or rootstock is critical as to whether grafts succeed or fail. A broadly expressed stele-specific promoter such as *pATHB8* might thus have an ability to perturb a greater degree of auxin response. In support of the notion that both rootstock and scion responses are relevant, mutating DOFs necessary for cambial divisions in either the rootstock or scion perturbs grafting (Zhang et al. 2022). A role for cambium in grafting has been long speculated including the advice to align cambiums for successful grafting (Aloni et al. 2010; Garner and Bradley 2013). Thus, our findings that cambium is important for both attachment and vascular formation contribute to deepening our understanding of the biology of grafting and the role auxin plays in promoting cell divisions and cell differentiation.

## Materials and methods

### Cloning

Plasmids were made using modules of Greengate cloning (Lampropoulos et al. 2013). For entry modules, the CDS of *bdl* was amplified from cDNA of the Arabidopsis (*A. thaliana*) *bdl-2* mutant (Col-0 background) or *iaa18-1* mutant (L*er* background) (Supplementary Table S1), then inserted into pGGC000 to create pGGC-*bdl* or pGGC-*iaa18*. The restriction and ligation reactions were done using BsaI-HF and T4 ligase (NEB), respectively. The resulting plasmids were transformed into *Escherichia coli* using chemically competent cells (Subcloning Efficiency DH5α Competent Cells, ThermoFisher Scientific) and extracted using Plasmid DNA Miniprep Kit (ThermoFisher). The final plant transformation vector pTRPY-*bdl* or pTRPY-*iaa18* was created by Greengate reaction as previously described (Lampropoulos et al. 2013) using pGGA016 (pOP plant promoter), pGGB003 (B-dummy), pGGC-*bdl*/pGGC-*iaa18* (*bdl* or *iaa18* CDS), pGGD002 (D-dummy), pGGE009 (UBQ10 terminator), pGGF008 (pNOS::Basta resistance::tNOS), and pGGZ003 (empty destination vector). The ligation product was used for *E. coli* transformation and the plasmid sequence confirmed by digestion analysis and sequencing the ligation sites. The plasmid was then co-transformed with helper plasmid pSOUP in *Agrobacterium tumefaciens* strain GV3101 and effector lines generated by introducing pTRPY-*bdl* or pTRPY-*iaa18* into Arabidopsis Col-0 using the floral dip method. The T_3_ homozygous transgenic plants were selected on ½ Murashige and Skoog (MS) media, 1% (w/v) plant agar, pH 5.8, containing 7.5 mg/mL Basta (glufosinate ammonium), and used for subsequent experiments.

### Plant materials, growth conditions, and grafting

Arabidopsis Columbia (Col-0) background was used throughout this study unless otherwise indicated. Seeds of driver lines (Schürholz et al. 2018) were obtained from NASC including *pAPL* (N2107943), *pATHB8* (N2107955), *pCASP1* (N2107953), *pLTP1* (N2107946), *pML1* (N2107954), *pPXY* (N2107941), *pSCR* (N2107940), *pSMXL5* (N2107942), *pTMO5* (N2107952), *pWOX4* (N2107945), and *pXPP* (N2107938). Mutant lines used included *bdl-2* (Hayward et al. 2009), *pRPS5A::GR:bdl* (Weijers et al. 2006), *iaa18-1* (Ploense et al. 2009), *athb8-11* (Prigge et al. 2005), *athb8-11,cna-2,phb-13,phv-11* (*bvca*) (Carlsbecker et al. 2010), *scr-3* (Fukaki et al. 1998), *slr-1* (Fukaki et al. 2002), *shr-2* (Fukaki et al. 1998), *wox4-1* (Hirakawa et al. 2010)*, pxy-3,wox4-1*, *pxy-3,pxl1-2,pxl2-1* (Etchells et al. 2013), and *smxl4,smxl5* (Wallner et al. 2017). The *athb8-11* and *bvca* mutants were in the Col *er*-2 background. The F_1_ seeds were obtained by crossing the *bdl* or *iaa18* effector lines to the driver lines expressing a synthetic transcription factor GR-LhG under various tissue-specific promoters harboring *mTurquoise2* fluorescent reporter. In the resulting F_1_ seedlings, GR-LhG drove the expression of *bdl* or *iaa18* as well as the *mTurquoise2* reporter when induced by DEX. For in vitro growth assays, sterile seeds were sown and grown on ½ MS media, 1% plant agar, pH 5.8. After stratification in the dark at 4°C overnight, the seeds were transferred to 20°C short-day growth conditions (8 h of 140 *μ*mol m^−2^ s^−1^). For growth assays, F_1_ seeds were sown and germinated without DEX for 5 d, and seedlings were transferred to media containing 5 *μ*m DEX for 5 d (Sigma-Aldrich). Confocal validation of tissue-specific expression of *bdl* and driver lines in the targeted tissues was performed with 7-d-old seedlings after 2 d of DEX induction. Arabidopsis grafting was performed on 5 to 7-d-old seedlings using previously published protocols (Melnyk et al. 2015). For DEX induction, 5 *μ*m DEX or equal volume of DMSO (Fisher Scientific) or EtOH was used instead of water in the grafting setup. Tissue attachment, phloem reconnection, and xylem reconnection were measured using previously published protocols (Melnyk et al. 2015).

### Cell proliferation detection

EdU staining was performed using Click-iT EdU Cell Proliferation Kit (Invitrogen). In brief, 10 *μ*m EdU was applied to grafted plants (3 DAG) for 1 h followed by tissue fixation, permeabilization, and staining following the manufacturer's instruction. Visualization of EdU was performed with confocal microscopy (Zeiss LSM780).

### Confocal imaging

Graft junctions were imaged using a Zeiss LSM780 NLO laser scanning confocal microscope. The tissues were cleared and stained with Calcofluor White staining protocol (Ursache et al. 2018), and visualized on the LSM780 with 405 nm excitation, 5% laser power, 410 to 529 nm emission, and 210 PMT. The mTurquoise2 signals were detected with 458 nm excitation, 5% laser power, and 460 to 516 nm emission. Vascular diameter quantifications included cambium, xylem, phloem, and pericycle tissues, and measured the distance between the pericycle layers encompassing the vascular bundle 50 or 100 *μ*m above or below the cut site. The images were processed and analyzed using Fiji software.

### Statistical analyses

Fisher's exact test was used for pairwise comparisons of frequencies with the indicated sample sizes. For pairwise comparisons of continuous data, unpaired 2-tailed Student's *t*-test was performed. One-way ANOVA analyses were performed with Prism 10.

### Accession numbers

The Arabidopsis Genome Initiative numbers of genes used in this study can be found in Supplementary Table S2.

## Supplementary Material

kiae257_Supplementary_Data

## Data Availability

The data underlying this article are available in the article and in its online supplementary material.

## Supplementary data

The following materials are available in the online version of this article.

**Supplementary Figure S1.** Auxin response is important for vascular reconnection and wound-induced callus formation.

**Supplementary Figure S2.** Perturbing cell-specific auxin responses modifies hypocotyl and root phenotypes.

**Supplementary Figure S3.** Auxin response is important for vascular reconnection and wound-induced callus formation.

**Supplementary Figure S4.** Perturbing cell-specific auxin responses modifies hypocotyl and root phenotypes.

**Supplementary Figure S5.** Perturbing auxin responses in the phloem inhibits successful graft formation.

**Supplementary Table S1.** List of primers used.

**Supplementary Table S2.** Arabidopsis Genome Initiative numbers of genes used or mentioned in this study.

## Dive Curated Terms

The following phenotypic, genotypic, and functional terms are of significance to the work described in this paper:

SCR Gramene: AT3G54220
SCR Araport: AT3G54220

IAA18 Gramene: AT1G51950
IAA18 Araport: AT1G51950

SHR Gramene: AT4G37650
SHR Araport: AT4G37650

WOX4 Gramene: AT1G46480
WOX4 Araport: AT1G46480

SMXL5 Gramene: AT5G57130
SMXL5 Araport: AT5G57130

BDL Gramene: AT1G04550
BDL Araport: AT1G04550

APL Gramene: AT1G79430
APL Araport: AT1G79430

CASP1 Gramene: AT2G36100
CASP1 Araport: AT2G36100

ATHB8 Gramene: AT4G32880
ATHB8 Araport: AT4G32880

LTP1 Gramene: AT2G38540
LTP1 Araport: AT2G38540

ML1 Gramene: AT4G21750
ML1 Araport: AT4G21750

PXY Gramene: AT5G61480
PXY Araport: AT5G61480

SLR1 Gramene: AT4G14550
SLR1 Araport: AT4G14550

TMO5 Gramene: AT3G25710
TMO5 Araport: AT3G25710

XPP Gramene: AT4G30450
XPP Araport: AT4G30450
