# Supplementary material for: Auxin signaling in the cambium promotes tissue adhesion and vascular formation during Arabidopsis graft healing
Source: Plant Physiol. 2024 May 3;196(2):754–62. doi: 10.1093/plphys/kiae257 (PMC11444275; doi:10.1093/plphys/kiae257)
Supplement: kiae257_Supplementary_Data [file kiae257_supplementary_data.zip › Serivichyaswat_supplementary_figures.pdf]

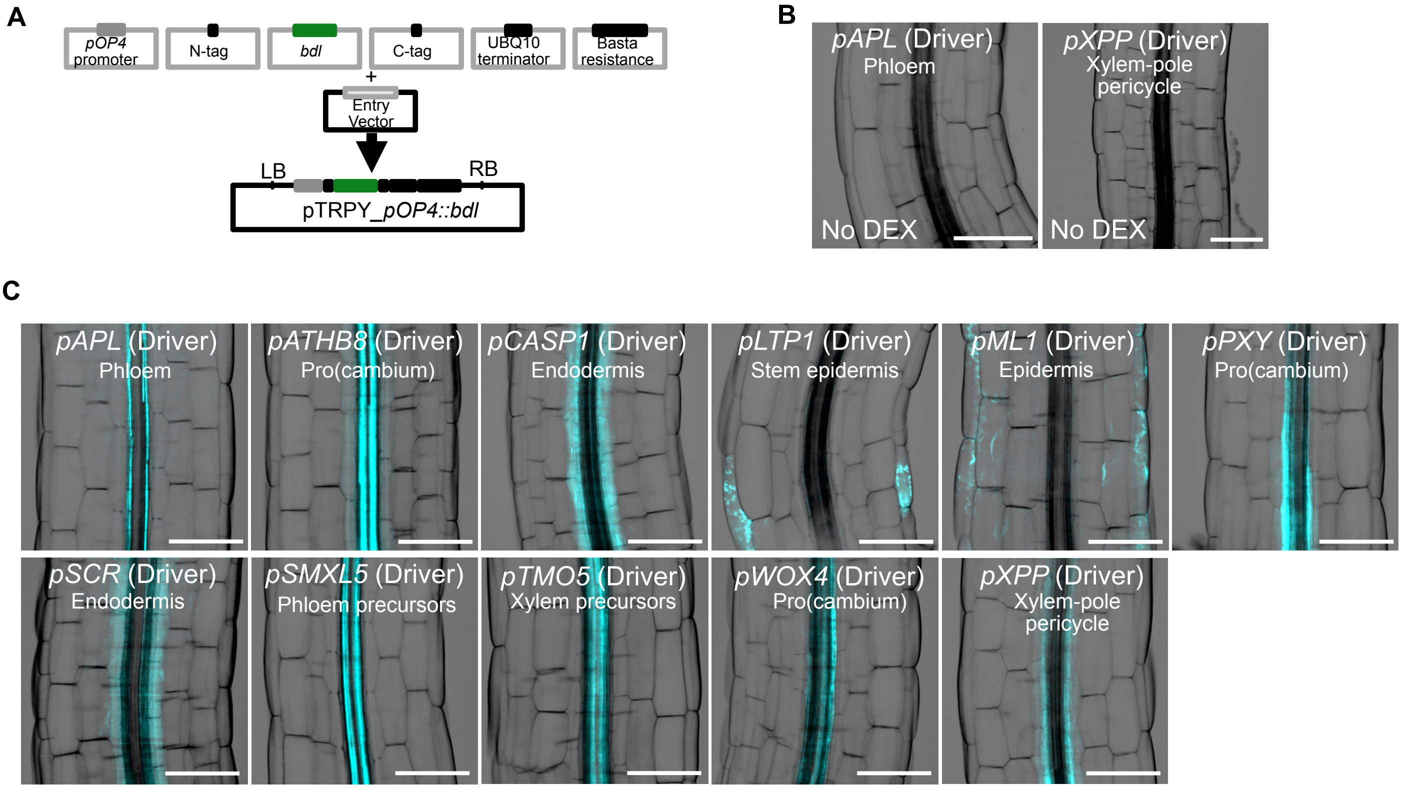

Supplementary Figure S1. Auxin response is important for vascular reconnection and wound induced callus formation. (A) The *bdl* coding sequence was cloned into the Greengate cloning system. Six entry plasmids, including a synthetic *pOP* promoter, an empty N-terminal tag, a *bdl*-coding sequence, an empty C-terminal tag, a UBQ10-terminator, and a plant selectable marker Basta resistance cassette, were used for a Greengate reaction, yielding the plant transformation destination vector pTRPY_*pOP4::bdl*, which was subsequently used to generate a transgenic *bdl* effector line. Left border (LB), right border (RB). (B) Examples of non-DEX induced hypocotyls with pAPL or pXPP driver lines. (C) Validation of tissue-specific expression of the DEX-induced driver lines in the targeted tissues by confocal imaging and detection of mTurquoise2 signals (cyan) in longitudinal optional sections of the hypocotyl of seven-day-old seedlings. (B-C) The LUT for the calcofluor white signal (grey), which marked the cell boundary, was modified to an inverted LUT in the Fiji software for better contrast. Scale bar is 100 µm.

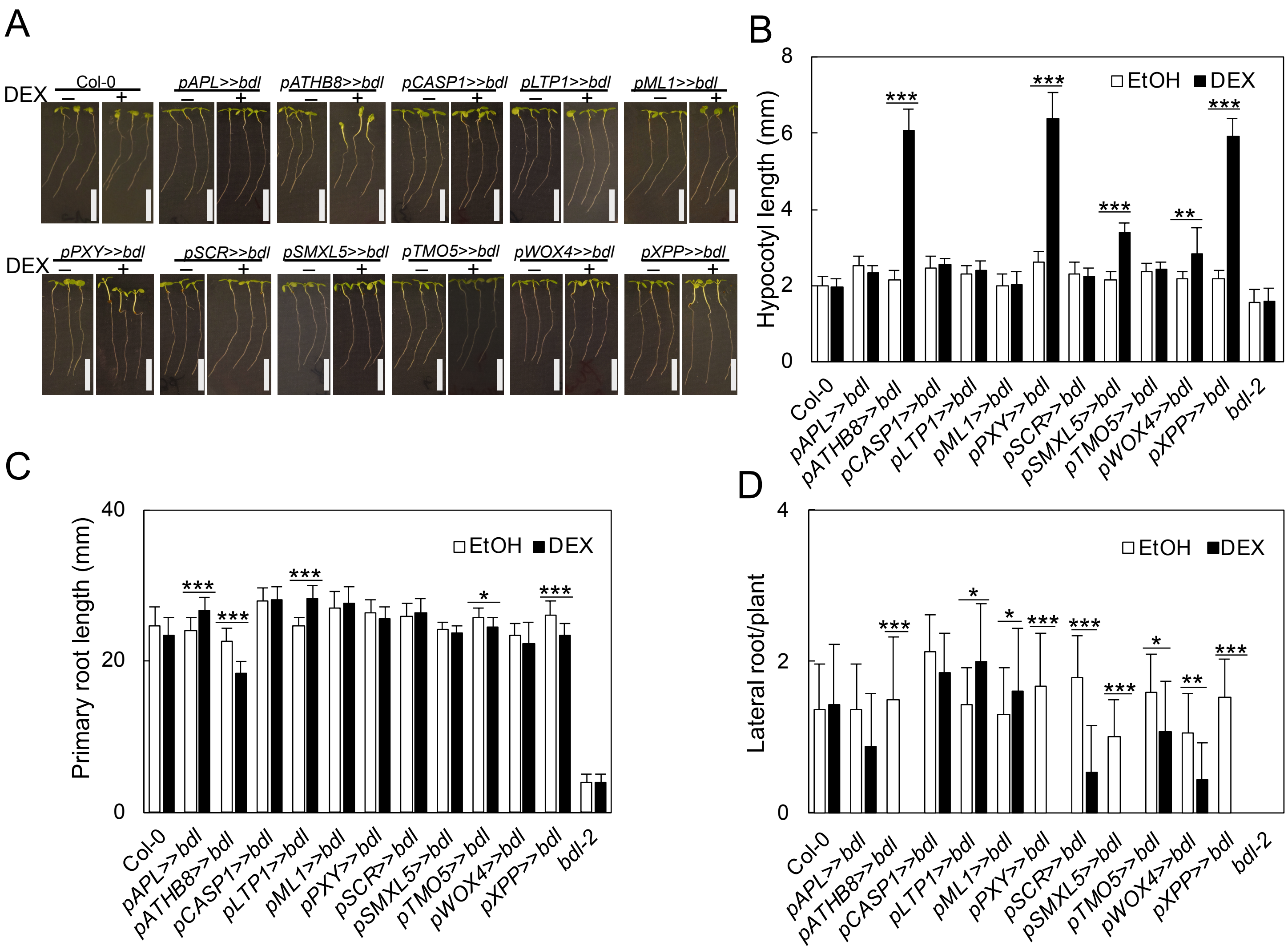

Supplementary Figure S2. Perturbing cell-specific auxin responses modifies hypocotyl and root phenotypes. (A-D) Phenotypic analysis of 10 day old F_1 plants expressing *bdl* in various tissues after 5 days dexamethasone (DEX) induction. Measurement of hypocotyl length (B), primary root length (C), and lateral root number (D) of transgenic plants expressing *bdl* under various tissue-specific promoters. Values represent mean±s.d. n=14-16 plants per genotype per treatment. *p<0.05; **p<0.01; ***p<0.001; student's t-test compared to mock controls. Scale bar is 1cm.

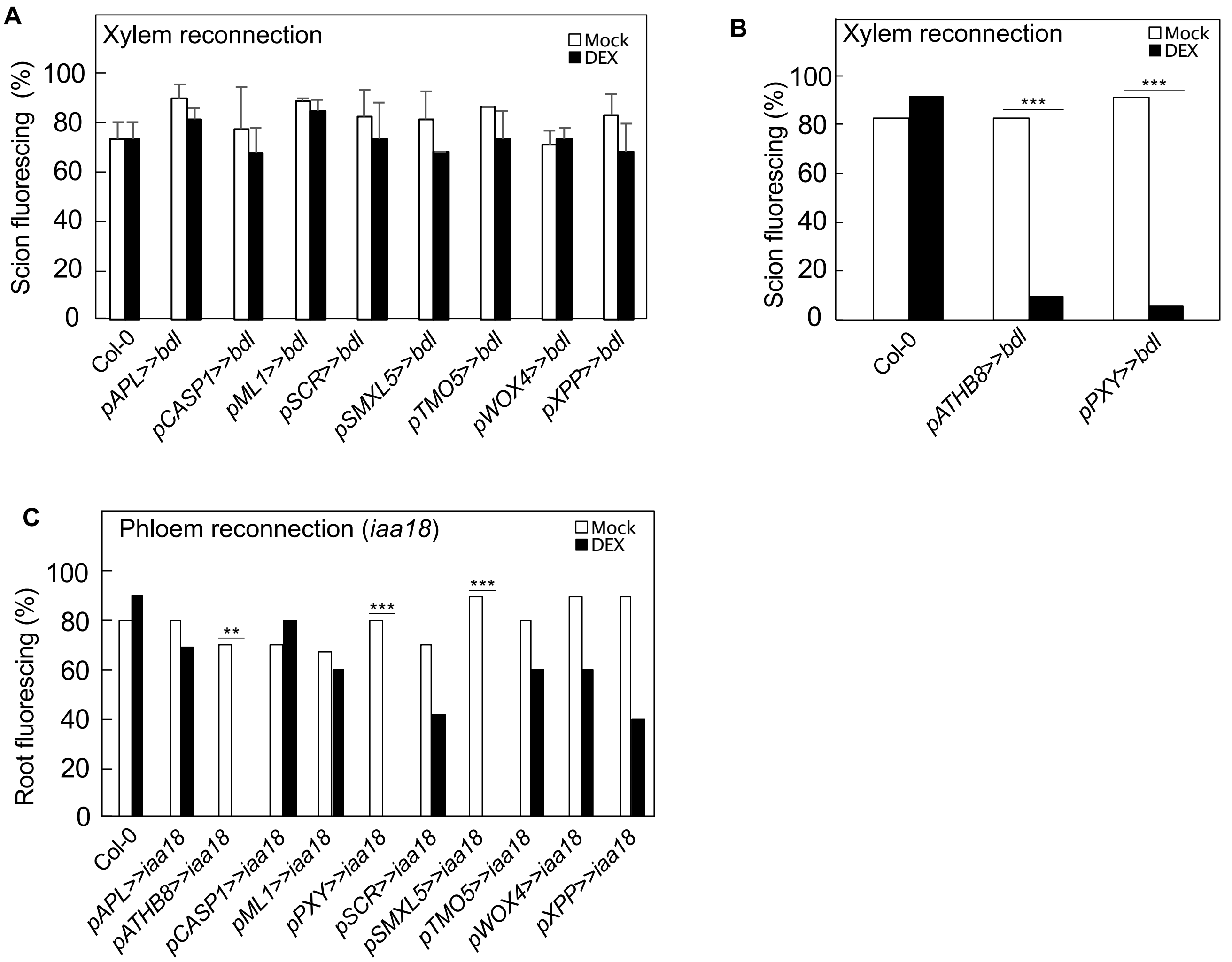

Supplementary Figure S3. Auxin response is important for vascular reconnection and wound induced callus formation. (A) Proportion of grafted transgenic Arabidopsis of selected genotypes that transported carboxyfluorescein diacetate (CFDA) to scions at seven days after grafting. Mean±s.d. n=20-25 plants per genotype per treatment. *p<0.05; **p<0.01; ***p<0.001; Fisher's exact test compared to mock controls. (B) Proportion of grafted *pATHB8>>bdl* or *pPXY>>bdl* that transported CFDA to the scion. Dexamethasone (DEX) was applied at 3 days after grafting and plants were sampled at 7 days after grafting. n=20-30 per treatment. *p<0.05; **p<0.01; ***p<0.001; Fisher's exact test compared to mock controls. C) Proportion of grafted Arabidopsis misexpressing *iaa18* that transported CFDA to the rootstock at 5 days after grafting. n=6-10 plants per genotype per treatment. *p<0.05; **p<0.01; ***p<0.001; Fisher's exact test compared to mock controls.

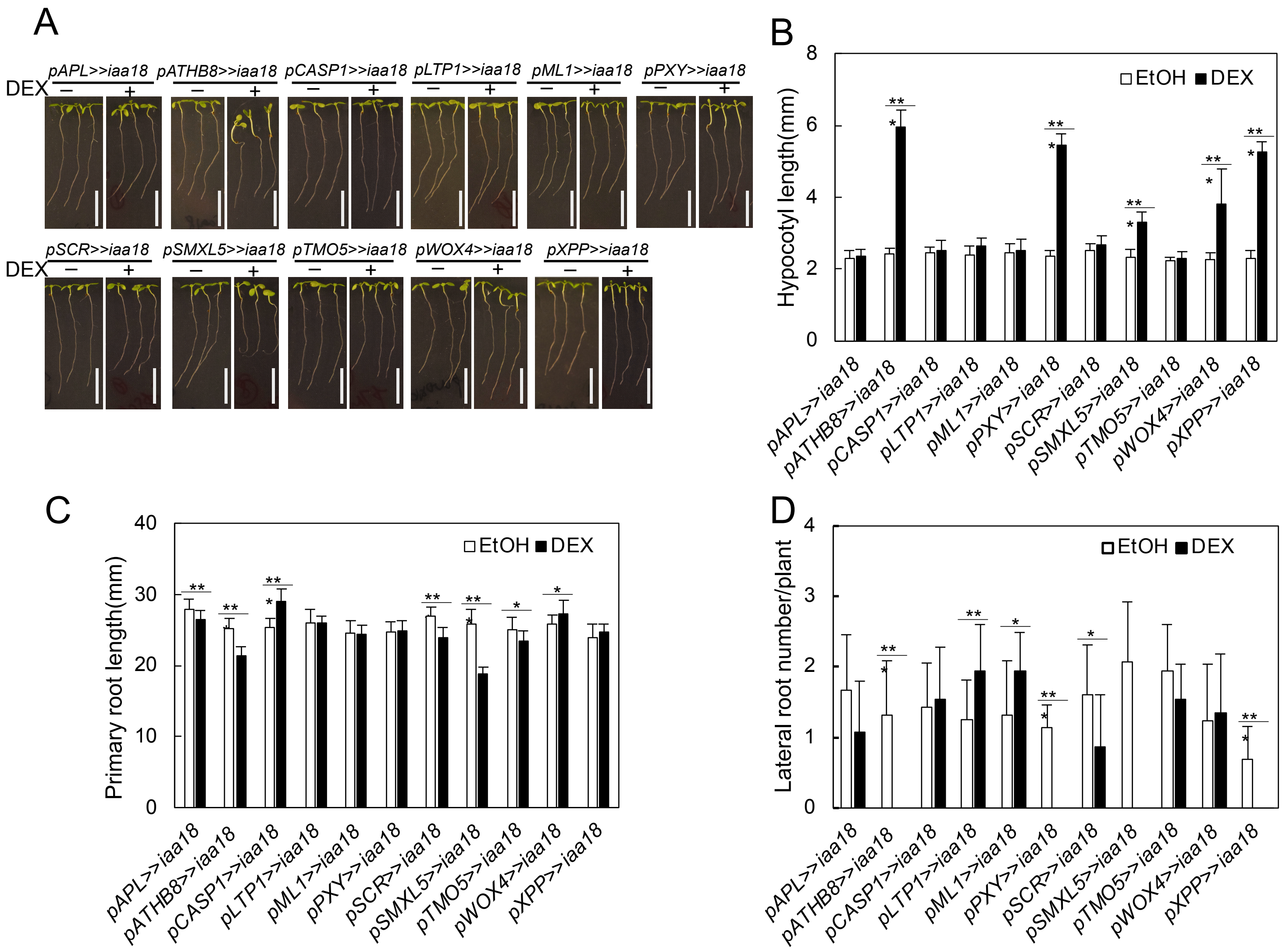

Supplementary Figure S4. Perturbing cell-specific auxin responses modifies hypocotyl and root phenotypes. (A-D) Phenotypic analysis of 10 day old F_1 plants expressing *iaa18* in various tissues after 5 days dexamethasone (DEX) induction. Measurement of hypocotyl length (B), primary root length (C), and lateral root number (D) of transgenic plants expressing *iaa18* under various tissue-specific promoters. Values represent mean±s.d. n=14-16 plants per genotype per treatment. *p<0.05; **p<0.01; ***p<0.001; student's t-test compared to mock controls. Scale bar is 1cm.

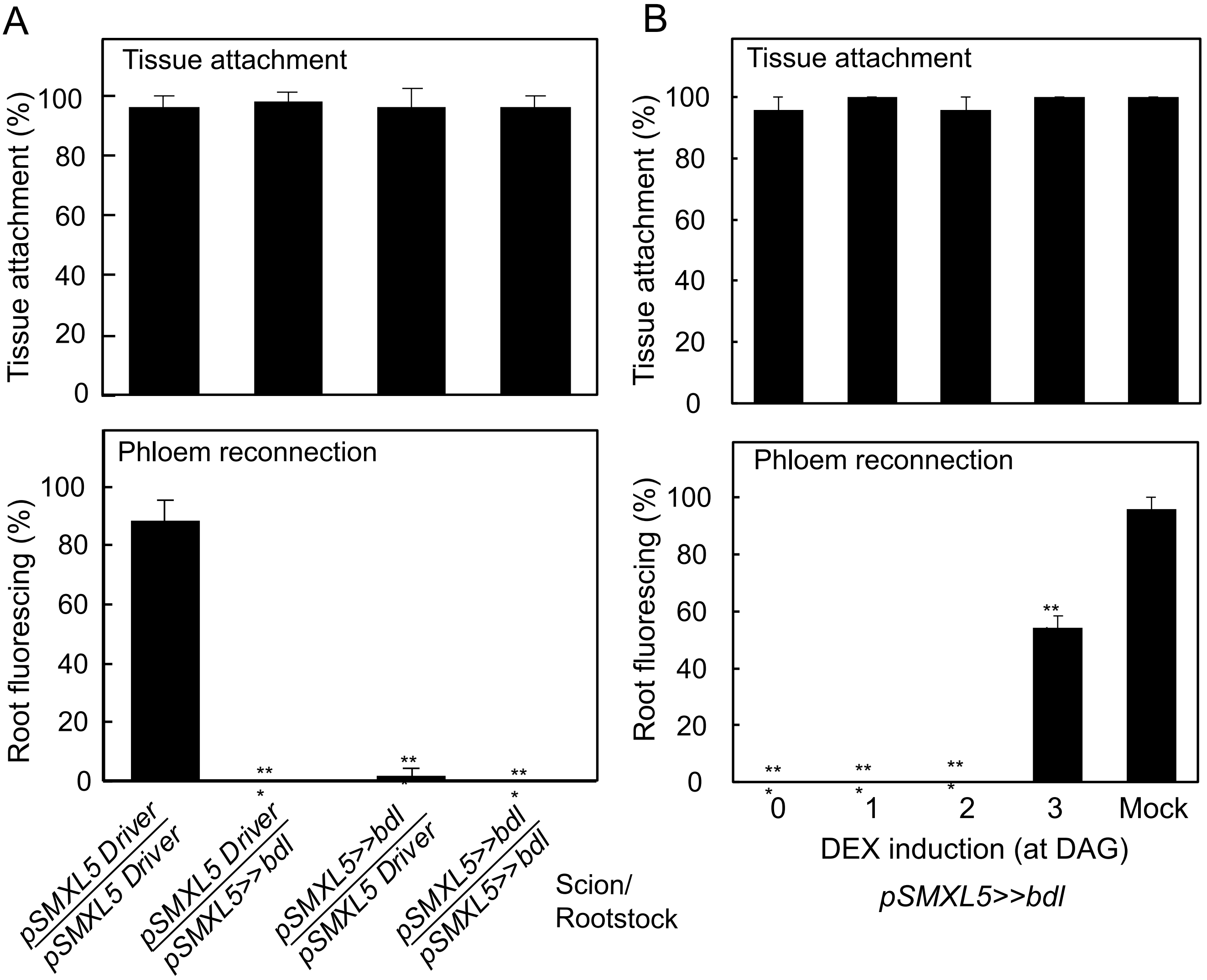

Supplementary Figure S5. Perturbing auxin responses in the phloem inhibits successful graft formation. (A) Proportion of dexamethasone (DEX)-treated and grafted *pSMXL5>>bdl* to the respective driver lines that attached or transported carboxyfluorescein diacetate (CFDA). The homo- and hetero-graft combinations are indicated. All plants were sampled at 5 days after grafting. Four replicates with 13-16 plants per treatment. Values represent mean±s.d. *p<0.05; **p<0.01; ***p<0.001, Fisher's exact test compared to mock or driver controls. (B) Proportion of grafts attached or that transported CFDA to the rootstock of grafted *pSMXL5>>bdl*. DEX was applied at indicated time points. Plants were sampled at 5 days after grafting. Two replicates with 12-16 plants per treatment. Values represent mean±s.d. *p<0.05; **p<0.01; ***p<0.001, Fisher's exact test compared to mock or driver controls.
