# Supplementary material for: Auxin signaling in the cambium promotes tissue adhesion and vascular formation during Arabidopsis graft healing
Source: Plant Physiol. 2024 May 3;196(2):754–62. doi: 10.1093/plphys/kiae257 (PMC11444275; doi:10.1093/plphys/kiae257)
Supplement: kiae257_Supplementary_Data [file pp.01741.2023-s04.pdf › pp.01741.2023-s04.pdf]

Plant Physiology®

Auxin signaling in the cambium promotes tissue adhesion and vascular formation during Arabidopsis graft healing

Phanu Serivichyaswat, Abdul Kareem, Ming Feng, and Charles Melnyk

Review Timeline:

Submission Date:	03-Nov-2023
Editorial Decision:	20-Dec-2023
Revision Received:	21-Feb-2024
Accepted:	08-Apr-2024

20-Dec-2023

Dr. Charles Melnyk
Sveriges lantbruksuniversitet
Uppsala
Sweden

RE: Auxin signaling in the cambium promotes tissue adhesion and vascular formation during Arabidopsis graft healing

Dear Dr. Melnyk:

We have received reviews of your manuscript, which you may view at the end of this letter. On the basis of the advice received and my own evaluation, I believe that, pending revision, this article may ultimately be acceptable for publication in Plant Physiology.

Both Reviewers have provided thorough and thoughtful comments to improve the clarity of the manuscript.

When you are ready to submit your revision, click the below link and find the red arrow under your Author Tasks heading for this revision submission.

If you want to visit this version of the manuscript, you may click the link below and then click on the folder that reads "Post Decision Manuscripts".

Link Not Available

When uploading the revised version of this article, please be sure to include as complete an answer as possible to the referees' criticisms during the first round of reviews in the "response to reviewers" section.

Plant Physiology considers a revision for up to 60 days after a manuscript has been provisionally accepted; limited extension of this timeframe is at the discretion of the Monitoring Editor. A reminder notice will be sent to you 2 weeks before the deadline, at which time you may choose to contact the editorial office to request an extension; if so, please provide a justification for your request. After the expiration of this deadline, the article would need to be submitted as a resubmission and would be handled as such.

Thank you very much for giving us an opportunity to review this work. I look forward to receiving the next version.

Sincerely,

Board of Editors, Plant Physiology

Want to add this revision deadline to your calendar? Click below!

-- Reviewer comments:
Reviewer #1 (Comments for the Author):

The authors use a tissue-specific inducible expression system to explore the role of auxin signaling in various tissue types during graft healing. They transformed Arabidopsis using the previously characterized bdl mutant which blocks auxin response factors, under a synthetic, promoter that expresses in the presence of a specific transcription factor. They then crossed this transformant with 11 existing lines that produce both the cognate transcription factor and the mTurquoise fluorescent protein, inducible by DEX application, and under tissue-specific promoters. These lines were verified by confocal microscopy following DEX induction, and seedling phenotypes were consistent with expectations based on previous auxin studies. The authors then grafted each of their bdl lines and found that bdl misexpression in the procambium-specific lines (pATHB8, and pPXY) failed at all aspects of grafting, including attachment, phloem reconnection and xylem reconnection, whereas misexpression in phloem

precursors (pSMXL5) and xylem pole pericycle (pXPP) prevented the reconnection of phloem. To ensure that these results were due to auxin deprivation (and not bdl-specific), they repeated the experiments using iaa18 in place of bdl. They then performed reciprocal grafting experiments on the procambium-specific bdl expression lines to determine the importance of auxin response in the scion and rootstock, and found that bdl expressed under pATHB8 reduced phloem connection in both, but only in the scion under pPXY. They also tested the effects of the timing of bdl in the procambium by inducing at 0-1, 1-2, and 3 days after grafting, and found that plants induced 0-1 DAG did not adhere, while 1-2 DAG did adhere but did not reconnect phloem, and those induced 3 DAG were unaffected. Next, the authors compared the localization of expression of the cambium specific (pATHB8 and PXY) and the phloem precursor (pSMXL5) driven lines by imaging the mTurquoise reporter both before and after induction, in grafted and in cut rootstocks and scions. In all cases, the signal was reduced after bdl induction. Finally, the authors used a stain that fluoresces during cell division and found a lack of cell division in plants expression bdl in the procambium.

Overall, this study provides new molecular evidence demonstrating that the procambium plays an important role in graft formation. This finding is not surprising, previous work has looked at the role of procambial patterning genes in grafting, but it does strengthen our understanding of the specific role of auxin in grafting formation. Several controls should be included in the paper, in addition to overall improvements in the writing.

The authors should include un-induced controls for all of their experiments evaluating the induced lines and investigate the tissue-specific expression of the induced target cell types in ungrafted stem tissue rather than root cell types, given that the relevant location for gene function during grafting is in the hypocotyl.

In Figure 4A-F, the cell type specificity of the mTurquoise reporter is not very clear. Higher resolution/higher magnification images should be provided for this experiment to accurately determine where the marker is expressed.

The introduction predominantly references the authors' papers, rather than properly citing research from the larger field of graft regeneration/tissue adhesion. It is essential to place the results within the context of known regenerative pathways, particularly the deep literature that is absent from the introduction on WIND1-induced regeneration.

The writing needs to be improved throughout the manuscript. There are numerous grammatical errors. For example - in the first paragraph I've inserted missing words in square brackets. This is an issue throughout the manuscript and should be addressed thoroughly by the authors: "Since ancient times, we have taken advantage of such regenerative abilities to join the shoot (scion) of one plant to the root (rootstock) of another, [through] a technique known as grafting (Garner & Bradley, 2013)." And "Grafting has allowed us to improve yields by combining superior properties such [as] plant size and stress tolerance into one plant (Melnyk, 2017)."

One minor suggestion:
Lines 58-59: "In addition, horticultural texts highlight the importance of correct cambial alignment for grafting success (Garner & Bradley, 2013)." There is very limited evidence to support this theory that correct cambial alignment is important for grafting success.

Reviewer #2 (Comments for the Author):

The authors report here the use of an elegant system for inducible tissue-specific expression and its application to characterize the effect of misexpression of a non-auxin-regulated form of the well-known BDL corepressor. The Introduction is quite complete and well-written. Materials and methods are very well described.
The system and driver lines utilized are shown in Figure 1, but the expression of two of the lines used, pAPL>>bdl and pLTP>>bld, is not shown there. The authors should include representative images of these two lines for completeness in Figure 1 and not as a Supplemental Figure. Also, some of the images shown are centered on the root tip, whereas the target tissue used by the authors to characterize the phenotype during graft formation is the central region of the hypocotyl. The images shown should always be focused on the target tissues being studied.
I see some inconsistencies in the seedling mutant phenotypes observed when bdl-2 was transactivated in different tissues.
(1) When bdl-2 was expressed using two procambium (pATHB8, pPXY) and one xylem pore pericycle (pXPP) promoters, the hypocotyl elongated significantly, but this was not the case when another procambium promoter (pWOX4) was used. What is the reason for this discrepancy?
(2) In previous experiments, expression of bdl using the pSHY2 promoter caused a strong reduction in hypocotyl elongation, confirming that bdl inhibits auxin responses required for hypocotyl elongation (https://doi.org/10.1038%2Fsj.emboj.7600659). Since BDL WT expression is normally restricted to the central vascular strands, the observed bdl-2 transactivation phenotype shown in this paper in the hypocotyl could be caused by a negative interaction with endogenous BDL, which could disrupt the normal turnover of downstream ARFs expressed in these tissues, including ARF6, NPH4/ARF7 and ARF8 (https://doi.org/10.1104/pp.18.00718). Hence, the interpretation of the phenotypes observed when BDL and bdl-2 expression overlap might be questionable. The characterization of the pBDL>>bdl-2 line needs to be included as a reference.
(3) Transactivation of bdl-2 in the epidermis leads to a partial phenocopy of the PR growth of bdl-2 mutants, but it has an opposite phenotype in terms of LR number, which increases. This is quite unexpected and difficult to reconcile with previous

reports on BDL function. How do the authors can explain this discrepancy?
(4) Transactivation of bdl-2 in different tissues (procambium; endodermis) leads to inconsistent results in terms of LR number: in some cases (pPXY, pWOX4; pSCR) LR decreases, in others not (pATHB8; pCASP1).
The results from Figure 3 are consistent with a dual role of BDL in the sequential steps of graft formation for tissue attachment, local cell proliferation, and phloem and xylem reconnection. bdl-2 expressed in the procambium was able to inhibit tissue attachment and subsequent phloem reconnection. Also, expression of bdl-2 in the phloem precursors was sufficient to inhibit phloem reconnection without altering tissue attachment. I have three comments on these results: (1) why did pWOX4>>bdl lines did not show any phenotype? (2) similar experiments as those shown for pATHB8 (C, D) and pPXY (E, F) should be shown here for pSMXL5, and (3) how to explain the rootstock-specific effect of bdl-2 misexpression with the pATHB8 promoter on phloem reconnection independent of tissue attachment (which seems normal under these conditions)?
Because of the relevance of the results shown in Supplemental Figure 2, I suggest to move this Figure to the main text. I have here the same remarks about the "cortex cells" (see my comment in minor changes).
I have no major concerns about the results shown in Figure 4, which clearly indicate that tissue attachment is required for DNA replication, as indicated by EdU staining. The legend states that "EdU staining detecting cell proliferation [...]", which is not quite accurate, as EdU staining is a marker for DNA replication, which precedes cell proliferation, but also cell endoreduplication.
The most striking thing about the results shown is the small reduction in tissue attachment of mutants defective in cambium (Figure 5), such as pxywox4 and pyxpxl1pxl2 (70% tissue attachment) compared to the results observed for bdl-2 transactivation in the same tissues (25% tissue attachment). Why did the authors not characterize the athb8 LOF mutants (e.g., mir165 lines; https://doi.org/10.1038/s41598-023-49093-2)? The results found for wox4 mutants and pWOX4>>bdl-2 are inconsistent. The explanation for the possible block in auxin signaling in the pWOX4 domain needs further elaboration.
In my opinion, the work presented here is very interesting, but needs some clarification.
Minor changes:
Change "Arabidopsis" to "A. thaliana" or "Arabidopsis thaliana". Alternative, use Arabidopsis without italics.
Line 85, change "different cell layers" to "different cell types".
Lines 165-165, EdU is a marker for DNA replication, not for cell division.
Line 240, "Ler", er needs to be in italics.
Line 248, change "Basta resistance" to "BastaTM resistance", for consistency with line 254.
Line 254, define 1/2MS (Murashige and Skoog).
Line 269, change "mTurquise2" to "mTurquoise2".
Fig. 1 legend. Change "30 um" to "30 M"; change mTurquise2 to mTurquoise2.
Fig. 2 legend. Change "tissue-specific promoter" to "tissue-specific promoters".
Fig. 3 legend. Change "values represent mean mean" to "values represent mean".
Fig. 4. The label for the "pink" stain (Calcofluor White) is not visible. Legend: AR is not shown in this figure. It is indicated that "asterisks indicate expanding cortex cells", but it cannot be excluded from the images that some of these cells may be from the epidermis instead.

We thank both reviewers for their comments. These suggestions have very much improved the manuscript and we are grateful for the time they put towards this.

Reviewer #1 (Comments for the Author):

The authors should include un-induced controls for all of their experiments evaluating the induced lines and investigate the tissue-specific expression of the induced target cell types in ungrafted stem tissue rather than root cell types, given that the relevant location for gene function during grafting is in the hypocotyl.

We have updated the manuscript to show both induced bdl-driver and induced driver lines with their expression patterns in the hypocotyl (revised Figure 1 and Supplemental Figure 1). We have also included representative non-induced ungrafted images from the hypocotyl (Supplemental Figure 1). Mock treatments have been relabeled as DMSO treated to indicate when un-induced controls were used. For confocal experiments where fluorescence is required, DEX treated driver lines were used as a control to see how fluorescence changes with bdl misexpression.

In Figure 4A-F, the cell type specificity of the mTurquoise reporter is not very clear. Higher resolution/higher magnification images should be provided for this experiment to accurately determine where the marker is expressed.

The healing graft junction is morphologically complex due to wounding, tissue damage, asymmetric division patterns and changes in cell identity. Even high resolution images of these graft junctions do not allow us to confidently assign where the marker is at the junction. See below for two examples from Figure 3A and Figure 3B that are visually striking but challenging to determine cell specificity based on morphology alone (pink is calcofluor white; mTurquoise is in blue; pATHB8 on the left, pPXY on the right).

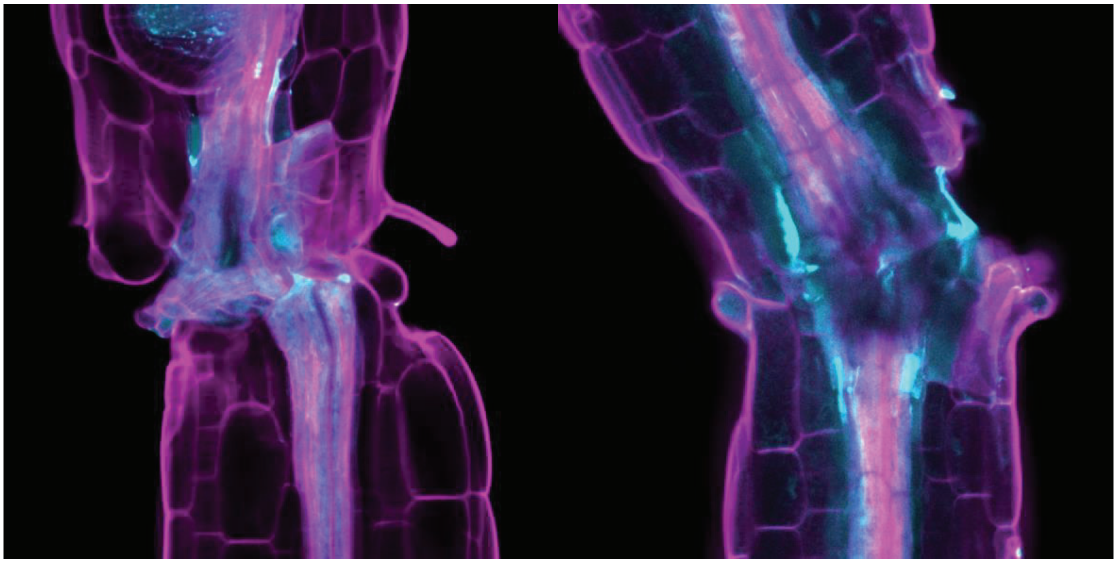

The introduction predominantly references the authors' papers, rather than properly citing research from the larger field of graft regeneration/tissue adhesion. It is essential to place the results within the context of known regenerative pathways, particularly the deep literature that is absent from the introduction on WIND1-induced regeneration.

Due to space constraints of the Report format, we are limited for the length of the introduction and are limited to ~30 references. We have added brief information about WIND1 in the introduction and also updated references in the introduction.

The writing needs to be improved throughout the manuscript. There are numerous grammatical errors. For example – in the first paragraph I've inserted missing words in square brackets. This is an issue throughout the manuscript and should be addressed thoroughly by the authors: "Since ancient times, we have taken advantage of such regenerative abilities to join the shoot (scion) of one plant to the root (rootstock) of another, [through] a technique known as grafting (Garner & Bradley, 2013)." And "Grafting has allowed us to improve yields by combining superior properties such [as] plant size and stress tolerance into one plant (Melnyk, 2017)."

We have updated the text to improve grammar.

One minor suggestion:
Lines 58-59: “In addition, horticultural texts highlight the importance of correct cambial alignment for grafting success (Garner & Bradley, 2013).” There is very limited evidence to support this theory that correct cambial alignment is important for grafting success.

We agree that the evidence is circumstantial and based on horticultural experience but nonetheless is mentioned in several texts or papers. For instance, it is written extensively in Garner and Bradley. See also these papers (among others) for reference to the perceived (but untested) importance of cambium and cambial alignment:

Rasool et al. 2020. Frontiers in Plant Science. 11.
Pina and Errea. 2005. Scientia Horticulturae. 106, 1-11.
Aloni et al. 2010. Scientia Horticulturae. 127, 119-126.

Reviewer #2 (Comments for the Author):

The system and driver lines utilized are shown in Figure 1, but the expression of two of the lines used, pAPL>>bdl and pLTP>>bld, is not shown there. The authors should include representative images of these two lines for completeness in Figure 1 and not as a Supplemental Figure.

Also, some of the images shown are centered on the root tip, whereas the target tissue used by the authors to characterize the phenotype during graft formation is the central region of the hypocotyl. The images shown should always be focused on the target tissues being studied.

We have updated Figure 1 to include APL and LTP1 images. Figure 1 now shows images from the hypocotyl. We have updated the supplemental section (Supplemental Figure 1) to show driver lines expression patterns from the driver misexpression lines to help the reader better understand the expression patterns in both uninduced, bdl and driver line systems.

I see some inconsistencies in the seedling mutant phenotypes observed when bdl-2 was transactivated in different tissues.

(1) When bdl-2 was expressed using two procambium (pATHB8, pPXY) and one xylem pore pericycle (pXPP) promoters, the hypocotyl elongated significantly, but this was not the case when another procambium promoter (pWOX4) was used. What is the reason for this discrepancy?

We have repeated this analysis and this time also included iaa18 misexpression lines (Supplemental Figures 2 and 4). The results are consistent in that pATHB8, pPXY and pXPP show the strongest effects with both bdl and iaa18. However, now we see a slight but significant effect with pSMXL5 and pWOX4, particularly with iaa18. We don’t know the specific reason why some promoters have a stronger effect than others, but believe it is not specific to bdl since we see a similar effect with iaa18. The differences in promoters could be due to differences in where they are expressed in the stele or due to promoter strength.

(2) In previous experiments, expression of bdl using the pSHY2 promoter caused a strong reduction in hypocotyl elongation, confirming that bdl inhibits auxin responses required for hypocotyl elongation (https://doi.org/10.1038%2Fsj.emboj.7600659). Since BDL WT expression is normally restricted to the central vascular strands, the observed bdl-2 transactivation phenotype shown in this paper in the hypocotyl could be caused by a negative interaction with endogenous BDL, which could disrupt the normal turnover of downstream ARFs expressed in these tissues, including ARF6, NPH4/ARF7 and ARF8 (https://doi.org/10.1104/pp.18.00718). Hence, the interpretation of the phenotypes observed when BDL and bdl-2 expression overlap might be questionable. The characterization of the pBDL>>bdl-2 line needs to be included as a reference.

bdl mutants show normal hypocotyl elongation (Hamann et al 1999) however elongation of the hypocotyl requires SHY2 dependent auxin responses. The BDL promoter is expressed in the stele, while the SHY2 promoter is induced in outer cell layers by auxin. Thus, we believe that the differences observed are likely due to differences in promoters. It’s possible that the bdl-2/BDL combination causes some ARF activation, but notably, Reed et al 2018 (https://doi.org/10.1104/pp.18.00718) show that *arf8* single mutants (and *arf6* to a lesser extent) increase hypocotyl lengths (Figure 4 in Reed et al). Thus, it’s possible that bdl and iaa misexpression in the stele might suppress the repressive effects of ARF6/7/8 and thus promote hypocotyl elongation. We have now included grafting assays with pRPS5A>>BDL-GR, a previously published homozygous line (Weijers et al 2006), showing that it too inhibits grafting (Figure 1) similar to our grafting assays with the misexpression lines (Figure 2) and arguing that a bdl-2/BDL combination is not what is blocking grafting.

(3) Transactivation of bdl-2 in the epidermis leads to a partial phenocopy of the PR growth of bdl-2 mutants, but it has an opposite phenotype in terms of LR number, which increases. This is quite unexpected and difficult to reconcile with previous reports on BDL function. How do the authors can explain this discrepancy?

We agree these results are hard to reconcile and have repeated these assays with bdl-2 and now also include iaa18 misexpression lines. In these updated data (Supplemental Figures 2 and 4), we don't see a strong effect of ML1 on primary root length, but still see a slight but significant increase in LR numbers with epidermal lines ML1 and LTP1 with iaa18 and bdl. LR numbers decrease in ATHB8, SMXL5, PXY, XPP and SCR for both iaa18 and bdl. For WOX4, we only see a decrease with bdl. Many ARF mutants reduce lateral root formation, so we believe our results are largely consistent with these previous data. The increase in lateral root numbers with both epidermal promoters with aux/iaa mutants is interesting but beyond the scope of this manuscript to study further.

(4) Transactivation of bdl-2 in different tissues (procambium; endodermis) leads to inconsistent results in terms of LR number: in some cases (pPXY, pWOX4; pSCR) LR decreases, in others not (pATHB8; pCASP1).
The results from Figure 3 are consistent with a dual role of BDL in the sequential steps of graft formation for tissue attachment, local cell proliferation, and phloem and xylem reconnection. bdl-2 expressed in the procambium was able to inhibit tissue attachment and subsequent phloem reconnection. Also, expression of bdl-2 in the phloem precursors was sufficient to inhibit phloem reconnection without altering tissue attachment. I have three comments on these results: (1) why did pWOX4>>bdl lines did not show any phenotype? (2) similar experiments as those shown for pATHB8 (C, D) and pPXY (E, F) should be shown here for pSMXL5, and (3) how to explain the rootstock-specific effect of bdl-2 misexpression with the pATHB8 promoter on phloem reconnection independent of tissue attachment (which seems normal under these conditions)?

We agree and have repeated the PR, LR and hypocotyl assays but this time with more plants, careful measurements and included iaa18. We believe the results are now more consistent. For the three points:

(1) This is interesting and may relate to where the WOX4 promoter is active or the promoter strength. We see an effect on LR numbers but much weaker than other cambial promoters. We discuss these possibilities in the discussion.
(2) Thanks for this suggestion. We agree and now include these data (Figure S5)
(3) Good point. We believe that cells contribute to at least two steps in graft formation: attachment and then later vascular formation/differentiation. ATHB8 affects both, perhaps through different cell types that are required for each step since ATHB8 has the broadest expression pattern in the stele. We have updated the discussion.

Because of the relevance of the results shown in Supplemental Figure 2, I suggest to move this Figure to the main text. I have here the same remarks about the "cortex cells" (see my comment in minor changes).
I have no major concerns about the results shown in Figure 4, which clearly indicate that tissue attachment is required for DNA replication, as indicated by EdU staining. The legend states that "EdU staining detecting cell proliferation [...]", which is not quite accurate, as EdU staining is a marker for DNA replication, which precedes cell proliferation, but also cell endoreduplication.

We have moved supplemental Figure 2 to the main text but due to space constraints, have moved the PR, hypocotyl and LR data to the supplemental. We have modified the figure legend as suggested.

The most striking thing about the results shown is the small reduction in tissue attachment of mutants defective in cambium (Figure 5), such as pxywox4 and pyxpxl1pxl2 (70% tissue attachment) compared to the results observed for bdl-2 transactivation in the same tissues (25% tissue attachment). Why did the authors not characterize the athb8 LOF mutants (e.g., mir165 lines; https://doi.org/10.1038/s41598-023-49093-2)? The results found for wox4 mutants and pWOX4>>bdl-2 are inconsistent. The explanation for the possible block in auxin signaling in the pWOX4 domain needs further elaboration.

We have now included the athb8 and bvca (*athb8-11 cna-2 phb-13 phv-11*) mutants. bvca is known to increase cambium and form more vascular cells (Carlsbecker et al 2010), consistent with the results we see in phloem assays. We agree regarding wox4 mutants and pWOX4>>bdl-2; however, wox4 may affect phloem reconnection independently of auxin. We also believe the WOX4>>bdl line to be quite weak in effects compared to other cambial lines (see the hypocotyl and lateral root data). The discussion has been updated.

In my opinion, the work presented here is very interesting, but needs some clarification.

Thanks for going over the text and figure legends carefully, this is appreciated. All suggestions below are taken or the text modified.

Minor changes:
Change "Arabidopsis" to "A. thaliana" or "Arabidopsis thaliana". Alternative, use Arabidopsis without italics. Changed
Line 85, change "different cell layers" to "different cell types". Changed
Lines 165-165, EdU is a marker for DNA replication, not for cell division. Modified
Line 240, "Ler", er needs to be in italics. Changed
Line 248, change "Basta resistance" to "BastaTM resistance", for consistency with line 254. Changed
Line 254, define 1/2MS (Murashige and Skoog). Changed

Line 269, change "mTurquise2" to "mTurquoise2". Changed
Fig. 1 legend. Change "30 um" to "30 μM"; change mTurquise2 to mTurquoise2. Changed
Fig. 2 legend. Change "tissue-specific promoter" to "tissue-specific promoters". Changed
Fig. 3 legend. Change "values represent mean mean" to "values represent mean". Changed
Fig. 4. The label for the "pink" stain (Calcofluor White) is not visible. Legend: AR is not shown in this figure. It is indicated that "asterisks indicate expanding cortex cells", but it cannot be excluded from the images that some of these cells may be from the epidermis instead. Changed

08-Apr-2024
Dr. Charles Melnyk
Sveriges lantbruksuniversitet
Uppsala
Sweden

MSID: PP2023-RR-01741R1
MS TITLE: Auxin signaling in the cambium promotes tissue adhesion and vascular formation during Arabidopsis graft healing

Dear Dr. Charles Melnyk:

I am pleased to inform you that your revised manuscript "Auxin signaling in the cambium promotes tissue adhesion and vascular formation during Arabidopsis graft healing" has been accepted for publication in Plant Physiology. Your manuscript will be checked for consistency with journal formatting and image standards by one of our Science Editors, who will contact you shortly. They may have a number of queries and suggestions for you. Please keep in mind that they work to increase the online visibility of your article and its attraction for readers from the widest of backgrounds. You will have the opportunity to review any revisions, and I hope you will work constructively with them to get the most out of your research. You will also have the opportunity to review all changes when you receive the author proofs for your article.

Once validated for publication, your article will be sent to a Science Editor for a final review of your manuscript and source files. After your final files are uploaded, your article will appear in the next available issue and the online Preview version of your article will be posted after your signed Copyright and Checklist forms and final source files are received.

Please be sure to include the following in your manuscript file:
Harvard style citations
List of all author contributions and funding information at the beginning of manuscript after the titles and author list.
List all supplemental materials associated with the article, with titles, at the end of the ms, before the references and acknowledgments section.
Complete Figure Legends for all figures appearing in the article listed before the Literature Cited.

Thank you and congratulations on your Plant Physiology paper!

Sincerely,
Board of Editors Plant Physiology

=====
FIRST AUTHOR PROFILES
=====

We recognize the hard work that goes into being First Author of a Plant Physiology paper by publishing a "First Author Feature" for each First Author alongside your paper.

If the first author(s) would like to submit information for a feature on Facebook and Twitter, fill out the form by clicking on the link below:

Plant Physiology First Author Feature Form: https://forms.office.com/r/ZBCGZfhm8F
If you cannot access the form through the link above, please contact Rachel at rbelsky@aspb.org.

To upload an image of yourself to accompany your profile, please put your photo in the correct journal folder at the link below. You must label your image: Author First Name Last Name Article Title.

First Author Profile Image Dropbox: https://www.dropbox.com/scl/fo/xz029p7qjr2jml2rp2kmq/h?dl=0&rlkey=6fkxcaqmxx35ap152b2osy1fz

=====
IMPORTANT REMINDER: PEER REVIEW REPORTS

=====

If you opted to publish a peer review report along with your article during the original submission process, it will be prepared by the editorial staff and publicly posted with your manuscript, inside the zip file that contains any other supplemental material. As a reminder, the peer review report is a public record of all comments from editors and reviewers, as well as your prior responses, as you received them in the decision letters for each draft of your manuscript. If you agreed to publish this report and have changed your mind, or are not sure if you selected this option, please contact the editorial office as soon as possible before signing the license agreement from our publisher.

-- Reviewer comments:
Reviewer #2 (Comments for the Author):

Thank you very much for considering my suggestions. I have no further comments.
